# Identifying tagging SNPs for African specific genetic variation from the African Diaspora Genome

**DOI:** 10.1038/srep46398

**Published:** 2017-04-21

**Authors:** Henry Richard Johnston, Yi-Juan Hu, Jingjing Gao, Timothy D. O’Connor, Gonçalo R. Abecasis, Genevieve L Wojcik, Christopher R. Gignoux, Pierre-Antoine Gourraud, Antoine Lizee, Mark Hansen, Rob Genuario, Dave Bullis, Cindy Lawley, Eimear E. Kenny, Carlos Bustamante, Terri H. Beaty, Rasika A. Mathias, Kathleen C. Barnes, Zhaohui S. Qin, CAAPA Consortium, Meher Preethi Boorgula, Monica Campbell, Sameer Chavan, Jean G. Ford, Cassandra Foster, Li Gao, Nadia N. Hansel, Edward Horowitz, Lili Huang, Romina Ortiz, Joseph Potee, Nicholas Rafaels, Ingo Ruczinski, Alan F. Scott, Margaret A. Taub, Candelaria Vergara, Albert M. Levin, Badri Padhukasahasram, L. Keoki Williams, Georgia M. Dunston, Mezbah U. Faruque, Kimberly Gietzen, Aniket Deshpande, Wendy E. Grus, Devin P. Locke, Marilyn G. Foreman, Pedro C. Avila, Leslie Grammer, Kwang-Youn A. Kim, Rajesh Kumar, Robert Schleimer, Francisco M. De La Vega, Suyash S. Shringarpure, Shaila Musharoff, Esteban G. Burchard, Celeste Eng, Ryan D. Hernandez, Maria Pino-Yanes, Dara G. Torgerson, Zachary A. Szpiech, Raul Torres, Dan L. Nicolae, Carole Ober, Christopher O Olopade, Olufunmilayo Olopade, Oluwafemi Oluwole, Ganiyu Arinola, Wei Song, Adolfo Correa, Solomon Musani, James G. Wilson, Leslie A. Lange, Joshua Akey, Michael Bamshad, Jessica Chong, Wenqing Fu, Deborah Nickerson, Alexander Reiner, Tina Hartert, Lorraine B. Ware, Eugene Bleecker, Deborah Meyers, Victor E. Ortega, Pissamai Maul, Trevor Maul, Harold Watson, Maria Ilma Araujo, Ricardo Riccio Oliveira, Luis Caraballo, Javier Marrugo, Beatriz Martinez, Catherine Meza, Gerardo Ayestas, Edwin Francisco Herrera-Paz, Pamela Landaverde-Torres, Said Omar Leiva Erazo, Rosella Martinez, Alvaro Mayorga, Luis F. Mayorga, Delmy-Aracely Mejia-Mejia, Hector Ramos, Allan Saenz, Gloria Varela, Olga Marina Vasquez, Trevor Ferguson, Jennifer Knight-Madden, Maureen Samms-Vaughan, Rainford J. Wilks, Akim Adegnika, Ulysse Ateba-Ngoa, Maria Yazdanbakhsh

**Affiliations:** 1 Department of Biostatistics and Bioinformatics, Emory University, Atlanta, GA, USA; 2 Data and Statistical Sciences, AbbVie, North Chicago, IL, USA; 3 Institute for Genome Sciences, University of Maryland School of Medicine, Baltimore, MD, USA; 4 Program in Personalized and Genomic Medicine, University of Maryland School of Medicine, Baltimore, MD, USA; 5 Department of Medicine, University of Maryland School of Medicine, Baltimore, MD, USA; 6 Department of Biostatistics, SPH, University of Michigan, Ann Arbor, MI, USA; 7 Department of Genetics, Stanford University School of Medicine, Stanford, CA, USA; 8 Department of Neurology, University of California, San Francisco, San Francisco, CA, USA; 9 Illumina, Inc., San Diego, CA, USA; 10 Department of Genetics and Genomics, Icahn School of Medicine at Mount Sinai, New York, NY, USA; 11 Department of Epidemiology, Bloomberg School of Public Health, Johns Hopkins University, Baltimore, MD, USA; 12 Department of Medicine, Johns Hopkins University, Baltimore, MD, USA.; 13 Department of Medicine, The Brooklyn Hospital Center, Brooklyn, NY, USA; 14 Department of Biostatistics, Bloomberg School of Public Health, JHU, Baltimore, MD, USA; 15 Department of Public Health Sciences, Henry Ford Health System, Detroit, MI, USA; 16 Center for Health Policy & Health Services Research, Henry Ford Health System, Detroit, MI, USA; 17 Department of Internal Medicine, Henry Ford Health System, Detroit, MI, USA; 18 Department of Microbiology, Howard University College of Medicine, Washington, DC, USA; 19 National Human Genome Center, Howard University College of Medicine, Washington, DC, USA; 20 Knome Inc., Cambridge, MA, USA; 21 Pulmonary and Critical Care Medicine, Morehouse School of Medicine, Atlanta, GA, USA; 22 Department of Medicine, Northwestern University, Chicago, IL, USA; 23 Department of Preventive Medicine, Northwestern University, Chicago, IL, USA; 24 Department of Pediatrics, Northwestern University, Chicago, IL, USA; 25 The Ann & Robert H. Lurie Children’s Hospital of Chicago, Chicago, IL, USA; 26 Department of Medicine, Northwestern Feinberg School of Medicine, Chicago, IL, USA; 27 Department of Bioengineering and Therapeutic Sciences, University of California, San Francisco, San Francisco, CA, USA; 28 Department of Medicine, University of California, San Francisco, San Francisco, CA, USA; 29 Institute for Human Genetics, Institute for Human Genetics, University of California, San Francisco, San Francisco, USA; 30 California Institute for Quantitative Biosciences, University of California, San Francisco, San Francisco, CA, USA; 31 CIBER de Enfermedades Respiratorias, Instituto de Salud Carlos III, Madrid, Spain; 32 Biomedical Sciences Graduate Program, University of California, San Francisco, San Francisco, CA, USA; 33 Department of Medicine, University of Chicago, Chicago, IL, USA; 34 Department of Statistics, University of Chicago, Chicago, IL, USA; 35 Department of Human Genetics, University of Chicago, Chicago, IL, USA; 36 Department of Medicine and Center for Global Health, University of Chicago, Chicago, IL, USA; 37 Department of Chemical Pathology, University of Ibadan, Ibadan, Nigeria, USA; 38 Department of Medicine, University of Mississippi Medical Center, Jackson, MS, USA; 39 Department of Physiology and Biophysics, University of Mississippi Medical Center, Jackson, MS, USA; 40 Department of Genetics, University of North Carolina, Chapel Hill, NC, USA; 41 Department of Genomic Sciences, University of Washington, Seattle, WA, USA; 42 Department of Pediatrics, University of Washington, Seattle, WA, USA; 43 University of Washington, Seattle, WA, USA; 44 Department of Medicine, Vanderbilt University, Nashville, TN, USA; 45 Department of Pathology, Microbiology and Immunology, Vanderbilt University, Nashville, USA; 46 Center for Human Genomics and Personalized Medicine, Wake Forest School of Medicine, Winston-Salem, NC, USA; 47 Genetics and Epidemiology of Asthma in Barbados, The University of the West Indies, Jamaica; 48 Faculty of Medical Sciences Cave Hill Campus, The University of the West Indies, Jamaica; 49 Queen Elizabeth Hospital, Queen Elizabeth Hospital, The University of the West Indies, Jamaica; 50 Immunology Service, Universidade Federal da Bahia, Salvador, BA, Brazil; 51 Laboratório de Patologia Experimental, Centro de Pesquisas Gonçalo Moniz, Salvador, BA, Brazil; 52 Institute for Immunological Research, Universidad de Cartagena, Cartagena, Colombia; 53 Instituto de Investigaciones Immunologicas, Universidad de Cartagena, Cartagena, Colombia; 54 Faculty of Medicine, Universidad Nacional Autonoma de Honduras en el Valle de Sula, San Pedro Sula, Honduras; 55 Facultad de Medicina, Universidad Catolica de Honduras, San Pedro Sula, Honduras; 56 Centro de Neumologia y Alergias, San Pedro Sula, Honduras; 57 Faculty of Medicine, Centro Medico de la Familia, San Pedro Sula, Honduras; 58 Tropical Medicine Research Institute, The University of the West Indies, Jamaica; 59 Department of Child Health, The University of the West Indies,. Jamaica; 60 Centre de Recherches Médicales de Lambaréné, Libreville, Gabon; 61 Institut für Tropenmedizin, Universität Tübingen, Germany; 62 Department of Parasitology, Leiden University Medical Center, Netherlands

## Abstract

A primary goal of The **C**onsortium on **A**sthma among **A**frican-ancestry **P**opulations in the **A**mericas (CAAPA) is to develop an ‘African Diaspora Power Chip’ (ADPC), a genotyping array consisting of tagging SNPs, useful in comprehensively identifying African specific genetic variation. This array is designed based on the novel variation identified in 642 CAAPA samples of African ancestry with high coverage whole genome sequence data (~30× depth). This novel variation extends the pattern of variation catalogued in the 1000 Genomes and Exome Sequencing Projects to a spectrum of populations representing the wide range of West African genomic diversity. These individuals from CAAPA also comprise a large swath of the African Diaspora population and incorporate historical genetic diversity covering nearly the entire Atlantic coast of the Americas. Here we show the results of designing and producing such a microchip array. This novel array covers African specific variation far better than other commercially available arrays, and will enable better GWAS analyses for researchers with individuals of African descent in their study populations. A recent study cataloging variation in continental African populations suggests this type of African-specific genotyping array is both necessary and valuable for facilitating large-scale GWAS in populations of African ancestry.

The design of the African Diaspora Power Chip (ADPC) was a primary goal as part of the NIH-supported **C**onsortium on **A**sthma among **A**frican-ancestry **P**opulations in the **A**mericas (CAAPA). Because of the overall poor coverage for African specific-variants on commercially available GWAS arrays, amongst other difficulties, relatively few GWAS have been performed in populations of African descent^1^,^2^, in part because they were underpowered to identify association with genes controlling risk for complex disease^3^. Previous GWAS studies in populations of African descent may have missed critical association signals because the single nucleotide polymorphisms (SNPs) genotyped on existing commercial arrays were selected for being informative among individuals of European ancestry, and generally do a poor job of tagging haplotypes and variants in individuals of non-European ancestry. This stems from the fact that the frequency of SNPs on currently available arrays is not well matched to the frequency of untagged variants in non-European populations. Essentially, the variant spectrum on current SNP arrays is flat, ensuring common genetic variants are well tagged, but making it difficult to tag low frequency SNPs (even though they may be highly polymorphic in non-European populations). This missing genetic variation in non-Europeans, however, consists largely of low frequency and rare variants, which will always be poorly covered by tagging SNPs with higher minor allele frequency (MAF). By building a large catalog of novel African-specific genetic variants, and then designing an array to tag as many of these as possible, we provide researchers with a significantly improved tool for hunting genes associated with diseases in populations of African ancestry, including admixed populations.

Ongoing work in the CAAPA consortium (Table S1, S2, Figure S1) has included coverage analysis of the novel variation identified by CAAPA sequencing. This analysis has shown that only 69% of common SNP variants and 41% of low-frequency SNP variants identified by CAAPA can be tagged by traditional GWAS arrays (at r^2^ >=0.8), such as the Illumina HumanOmni5 (which contains about five million SNPs)^4^. Ha, *et al*.^5^ suggested much lower coverage levels, with the OmniExpress chip (containing about 770,000 SNPs) effectively covering only 8% of known variation within the YRI genome, while the much larger Omni 2.5 (containing about 2.5 million SNPs) still only covers 20% of known YRI SNPs based on their analysis. In contrast, variants of European (CEU) ancestry are 21% covered by the OmniExpress and 44% covered by the Omni 2.5. These are large differences in genomic coverage, and are supported by other studies with similarly pessimistic estimates of effective coverage among non-European populations^6^,^7^,^8^. Even the primary manufacturer of the Omni series genotyping chips, Illumina, refers to low coverage levels for their currently available commercial GWAS arrays in non-European populations. Table 1 It is important to note that there is no standardized definition for efficient ‘coverage,’ as each method uses a different set of SNPs to assess coverage levels. Regardless of the method used, however, contemporary commercially available arrays do a poor job of tagging common haplotypes or ‘covering’ all genetic variation in non-European or admixed populations.

Usage of the most recent imputation panels can significantly improve the coverage of African variants, but this practice is still hamstrung by the lack of low-frequency variants on genotyping arrays (MAF < 5%). Imputation of low-frequency variants is most efficient and accurate when the SNP to be imputed has a similar minor allele frequency as the genotyped SNP, so a relative lack of low frequency variants on an array can render imputation of similar frequency variants difficult [Marchini and Howie, 2010].

To address this shortcoming, the ADPC was designed using the whole-genome sequencing results on 642 CAAPA samples, including 328 African Americans, 125 African Caribbean subjects, 164 African ancestry individuals with some Latino ancestry, and 25 individuals from Nigeria. The whole genomes of these individuals were sequenced using the Illumina HiSeq 2000. A total of 47.9 million biallelic SNPs were identified in these CAAPA samples. Of those, 15.6 million variants have a MAF greater than or equal to 1%.

To create an affordable array for large-scale chip-based studies, 700,000 variants was the maximum size of the array. A MAF of 1% was chosen as the preliminary cutoff to limit the initial pool of variants to be tagged. In addition, using 1% as the MAF cut-off eliminated concerns about potential false positive variant calls for rare variants derived from the sequence data^9^. Additionally, the ADPC is designed to be used in conjunction with the OmniExpress array, a low-cost GWAS array popular among researchers, leveraging the high MAF coverage available from OmniExpress and freeing the ADPC to focus on low frequency variants.

To narrow the pool further, SNPs with poor Illumina design scores (proprietary scores which attempt to give a numeric representation of the likelihood that a marker will work properly on the array) were removed from consideration as potential tag SNPs. MaCH^10^ and minimac^11^ were used to determine which CAAPA variants were well-imputable based on the 1000 Genomes Phase I African Reference Panel and variants from the OmniExpress array. Those SNPs that could be imputed well (r^2^ > 0.8) were removed from the pool of SNPs needing to be tagged.

Fugue^12^ was then used to determine the pairwise linkage disequilibrium (LD) between each pair of SNPs in the remaining set of SNPs. These LD estimates were used by FESTA^13^, a TagSNP selection program, to select SNPs using a rather strict r^2^ threshold of 0.8. A total of 1,004,268 TagSNPs were selected. Among them, 4,000 were removed because they were too similar in their probe design to function well on the array. Limited by the capacity of the array, only TagSNPs with an MAF >=1.6% were retained for inclusion on the array. Raising the MAF floor was deemed to be the best approach to thinning the TagSNP pool as it was unbiased. Additional content was then added including both SNPs previously found to be associated with African-specific diseases but not previously selected as TagSNPs and approximately 600 additional SNPs in the human leukocyte antigen (HLA) region. HLA SNPs added to the array are relevant for HLA imputation and analyses of diseases or phenotypes related to immunity. These SNPs represent a pruned selection of SNPs with high tagging power, directly through LD, as well as SNPs preferentially selected by existing SNP-based HLA imputation algorithms^14^. Finally, a “GWAS fingerprint” that includes 274 markers, identical to that used on the Illumina HumanExome array, was added to enable researchers to ensure accurate sample labeling and analysis, while facilitating sample tracking across experiments. In final count, 627,998 variants were included on the ADPC array Fig. 1.

## Results

**Figure 2:**
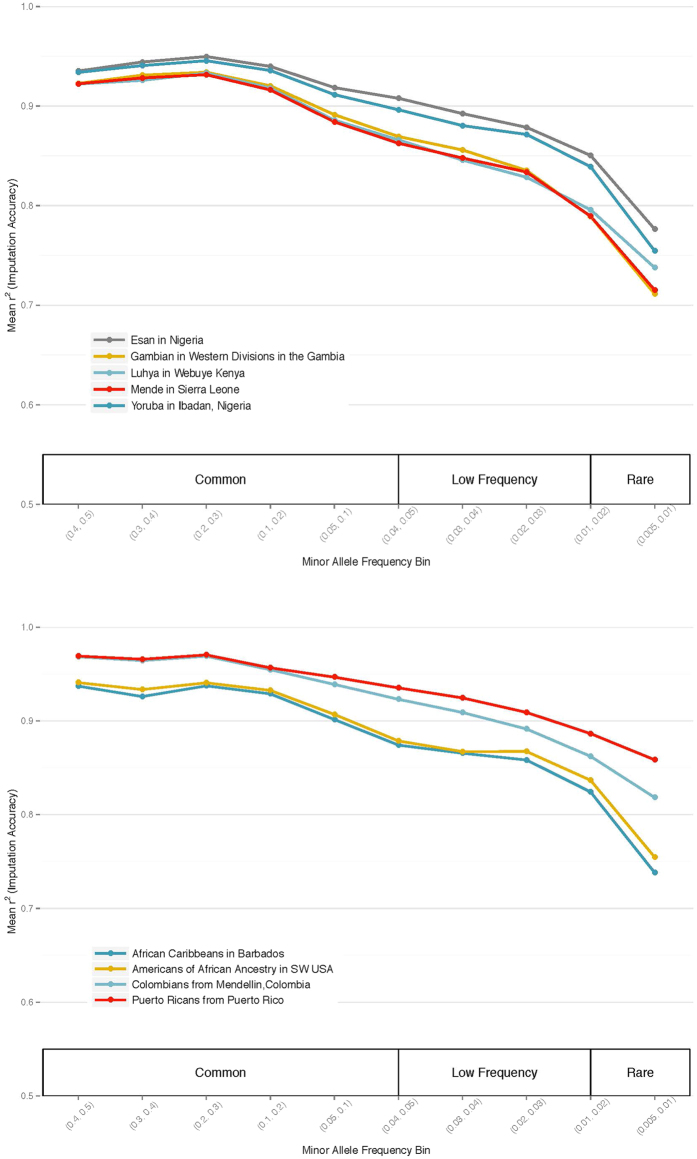
Estimated imputation coverage of variants tagged by the ADPC content as part of the MEGA array. (**a**) Coverage in 1000 Genomes African populations is >=0.8 r^2^ down to 1% MAF. (**b**) Coverage in 1000 Genomes admixed African populations is >=0.8 r^2^ down to 1% MAF

Based on the combination of OmniExpress and the ADPC, coverage is exceptionally good in both the 1000 Genomes African and admixed African populations. Figure 2 The average r^2^ for all variants is greater than 0.8 at ≥1% MAF. Coverage is slightly better amongst admixed African populations than continental African populations, which is useful for the study of African Americans in particular. It is also not surprising, given that we had only one continental African population, compared to 15 African-admixed populations. In all populations, this represents a much better coverage level than with previous commercially available arrays, and represents an important step forward for studies of individuals of African descent.

Additional analysis of SNP coverage at ≥ 1% MAF in the CAAPA population was conducted to ensure our array will enable researchers to have sufficient power to identify novel associations between disease phenotypes and low frequency variants specific to African populations. Despite raising the MAF threshold for TagSNPs to 1.6%, we report coverage of all variants with MAF greater than or equal to 1% to give a full picture of the low frequency coverage the ADPC can provide. Genome-wide, the OmniExpress array is estimated to tag 20% of CAAPA variants at *r*^2^ = 0.9, 26% at *r*^2^ = 0.8 and 39% at *r*^2^ = 0.5. All selected variants of the ADPC, alone, are estimated to tag 12% of known variants at *r*^2^ = 0.9, 16% at *r*^2^ = 0.8, and 31% at *r*^2^ = 0.5. The combination of these two arrays is estimated to tag 29% of all CAAPA variants at *r*^2^ = 0.9, 37% at *r*^2^ = 0.8, and 56% at *r*^2^ = 0.5, an improvement of about 50% more variants tagged over the OmniExpress array across the three thresholds Table 2.

While we consider these coverage statistics strong, they only refer to the coverage for variants identified through whole-genome sequencing in CAAPA. This is the most difficult possible test set, since coverage of more common variants in the 1000 Genomes data is not included here. We use this information to give researchers an accurate view of the coverage available for the wealth of novel, low frequency genetic variation identified by CAAPA’s whole-genome sequencing.

Using ~12,000 samples from the CAAPA consortium for the initial run of the ADPC array, ~495,000 out of 700,000 variants passed Illumina’s QC thresholds. This relatively high marker failure rate is not unexpected, however, because the array is comprised of nearly 100% novel markers, never before manufactured. The 494,094 markers successfully manufactured performed excellently, with missing genotype rate averaging only 0.3%.

**Figure 3:**
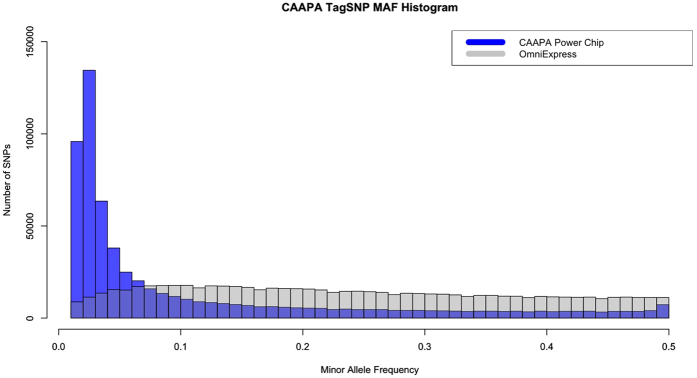
Projected minor allele frequency histograms for the ADPC and OmniExpress arrays overlayed with one another. The disparity between the arrays is significant, and represents very different tagging approaches. This makes them well suited to complement each other.

An important and unique feature of the ADPC is the significantly skewed MAF spectrum of variants on the array Fig. 3. Compared to OmniExpress, the ADPC contains vastly more low frequency variants (Figure S2). This was not a conscious decision in the design process. Instead, it is the result of trying to tag a set of variants not previously tagged by commercially available genome-wide marker arrays. As a result, the combination of the ADPC and OmniExpress is an efficient pairing that increases coverage of the full MAF spectrum for novel variants and dramatically improves the imputation power for low frequency variants.

The ADPC content is currently available as part of the MEGA array through Illumina. That array combines the APDC content described here plus a GWAS backbone based on OmniExpress, plus additional content from around the world to provide a single use array for researchers. Through the use of this array, researchers will have greater statistical power to find associations with complex diseases in populations of African ancestry, which has several practical benefits. This array will provide additional value from its tremendous improvement in the quality of imputed genotypes across the genome. At the same time, new researchers will be able to determine power before starting a study in populations of African ancestry, using the combination of the ADPC and OmniExpress on the MEGA array, leading to smaller sample sizes needed, and more studies being possible.

## Discussion

In this paper, we present the African Diaspora Power Chip, an affordable genotyping array that dramatically increases the coverage of genetic variants specific to African populations (and their descendants). Through the use of this array, researchers can now be better powered to detect disease associations in populations of African ancestry. For the CAAPA consortium, this means using the ADPC to genotype >13,000 Asthma cases and controls from 9 populations across the Americas.

Although this array was designed to meet the specific needs and timeline of the CAAPA consortium, and may not represent the ideal for a strictly African-based SNP array, its content is available to researchers now as part of the MEGA array and has demonstrably increased coverage in variants of West African ancestry. This is, of course, the most common African admixed population in African Americans or other members of the African diaspora, so it is especially well positioned to be useful in studies of admixed African Americans. This population, which has been under-covered by previously existing arrays should benefit greatly from the SNP content now available. Furthermore, the CAAPA consortium has released an imputation reference panel, based on the results of our whole-genome sequencing experiment, and it is available through the Michigan Imputation Server (https://imputationserver.sph.umich.edu/index.html), and maximizes coverage provided by the ADPC content.

Our immediate plans are to assess the coverage provided by the ADPC content in populations of African descent not originating in West Africa. Of specific interest to geneticists are populations in East and North Africa. In the future, through the use of the MEGA array, the number of meaningful results from GWAS studies conducted in populations of African descent should increase significantly, providing a more accurate picture of causal disease variants in this group. This will enable personalized medicine techniques to be applied to a much larger subset of Americans than is currently feasible.

## Methods

All study participants in the whole genome sequencing study provided written informed consent for the use of their DNA in genetic studies. A careful review was conducted to verify that the consents, study methods, and experimental protocols were consistent with the activities of this study. All methods were performed in accordance with the relevant guidelines and regulations. Institutional review board approval was obtained at Johns Hopkins University (GRAAD, BAGS, BIAS, HONDAS, PGCA), Howard University (GRAAD), Columbia University (REACH), Wake Forest University (SARP), Morehouse School of Medicine (COPDGene), Henry Ford Health System (SAPPHIRE), the University of California, San Francisco (coordinator center for SAGE II and GALA II), the Western Institutional Review Board for the recruitment in Puerto Rico (GALA II Puerto Ricans), Baylor College of Medicine from Texas, Albert Einstein College of Medicine Yeshiva University, Jacobi Medical Center, the North Central Bronx Hospital from New York (GALA II Dominicans), Children’s Hospital and Research Center Oakland and Kaiser Permanente-Vallejo Medical Center (SAGE II), Vanderbilt University (BREATHE, VALID), the University of Chicago (CAG, AEGS), University of the West Indies, Mona campus (JAAS) and Cave Hill Campus, Barbados (BAGS), The University of Cartagena (PGCA), the Universidad Católica de Honduras in San Pedro Sula (HONDAS), the Federal University of Bahia and endorsed by the National Commission for Ethics in Human Research in Brazil (BIAS, SCAALA), and The University of Ibadan, Nigeria (AEGS).

The 642 individuals in the data freeze were sequenced using Illumina’s Hi-Seq 2000 equipment and the reads were 100 bp, paired-end runs. Assembly was performed by the Consensus Assessment of Sequence and Variation (CASAVA) package, which is the Illumina in-house assembly and variant calling technology. The SNP-caller implemented in CASAVA uses a probabilistic model to ultimately generate probability distributions over all diploid genotypes for each site in each genome. A set of MAXGT quality scores is thus generated for each genomic site, corresponding to the ‘consensus quality’ in the SamTools SNP calling method^15^. These quality scores are then parsed based on a set of consortium-wide rules in order to determine the likely set of variants.

Data processing to generate a 691-sample VCF file for each chromosome from the Illumina MAXGT single-sample SNP VCF files provided in Illumina’s standard deliverable package was performed at Knome, Inc. (Cambridge, MA, USA). The individual VCF files only contained calls for variants, not ref/ref homozygotes. To generate a multi-sample VCF file, these individual VCF files were merged using VCFTools^16^ (v0.1.11), then using custom scripts, a multi-sample VCF file was backfilled to include homozygous reference genotypes and depth of coverage from the sites.txt files. Custom QC scripts confirmed the multi-sample VCFs and the single-sample VCFs had the same number of heterozygous and homozygous alternate genotypes. VCFtools was used to confirm all subjects were included in each multi-sample VCF. The multi-sample VCF was generated including the 48 samples from the SCAALA (Salvador, Brazil) group, but these samples were subsequently dropped from all analyses, leaving 642 individuals, and variants unique to SCAALA were removed from the variant pool. Mathias *et al*.^4^.

To pare down the list of variants needing to be tagged, several exclusion sets were created, starting with design score analysis. The segment extending 60 base pairs up and down stream from each variant position were surveyed to determine which side of the variant would create a better probe and a design score was calculated, on a 0–1 scale, representing the estimated success rate for the variant. Any variant scoring below 0.5 was removed. Variants already on the OmniExpress array were also excluded.

To determine which CAAPA variants could be well imputed, the software packages MaCH^10^ and minimac^11^ were employed. All CAAPA samples were first pre-phased by MaCH; subsequently variants still remaining in the tagging pool were imputed in minimac, a low-memory, computationally efficient variant of MaCH, specifically designed for haplotype-to-haplotype imputation. Variants were imputed using the 1000 Genomes Phase I African Reference Panel as the reference.

## Additional Information

**Accession codes:** The whole genome sequence data that support the findings of this study have been deposited in dbGAP with the accession code phs001123.v1.p1.

**How to cite this article:** Johnston, H. R. *et al*. Identifying tagging SNPs for African specific genetic variation from the African Diaspora Genome. *Sci. Rep.* **7**, 46398; doi: 10.1038/srep46398 (2017).

**Publisher's note:** Springer Nature remains neutral with regard to jurisdictional claims in published maps and institutional affiliations.

## Supplementary Material

Supplementary Information

## Figures and Tables

**Figure 1:**
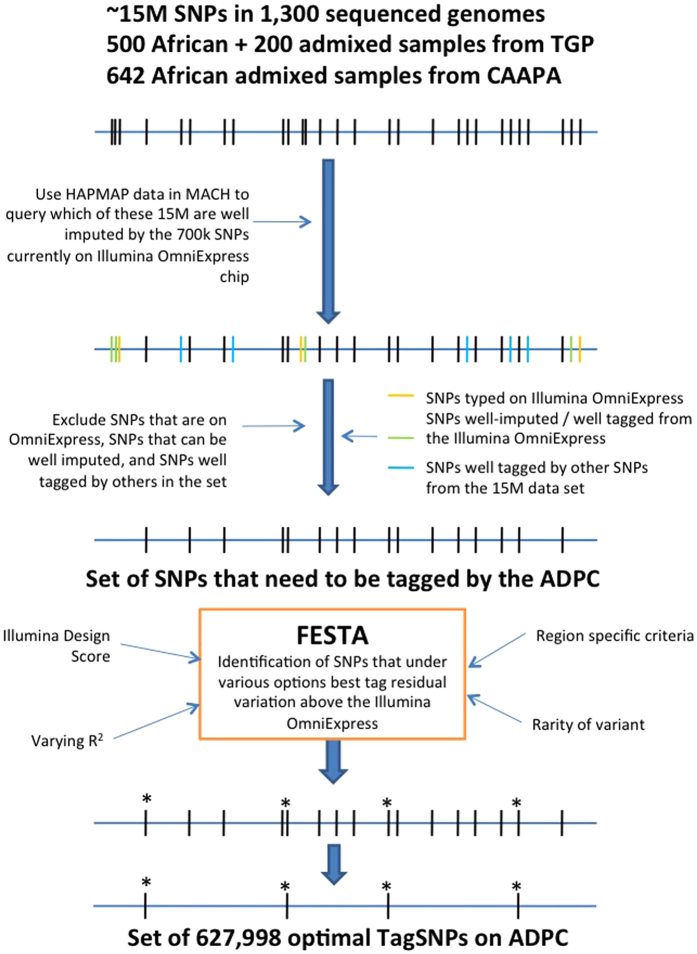
The ADPC design pipeline, describing the steps taken to whittle ~15 million novel African SNPs into a 627k African-targeted GWAS array.

**Table 1:** Illumina projected coverage of African variants on several commercially available GWAS arrays.

Array Type	MAF Category			
	<0.010 (non-singletons)	>0.010	>0.025	>0.050
HumanExomev1-2	0.031	0.032	0.034	0.035
HumanCore-12v1-0	0.127	0.152	0.203	0.256
HumanCoreExome-12v1-0	0.148	0.172	0.221	0.271
OmniExpress-12v1-1	0.210	0.249	0.326	0.395
OmniExpressExome-8v1-1	0.226	0.263	0.337	0.403

**Table 2:** Projected coverage for the ADPC among CAAPA variants >=1% MAF, with and without OmniExpress pairing, for the whole genome.

Coverage of CAAPA variants >=1% MAF			
r^2^	OmniExpress Alone	ADPC Alone	Combined
0.9	20%	12%	29%
0.8	26%	16%	37%
0.5	39%	31%	56%

## References

[b1] Gurdasani D. . The African Genome Variation Project shapes medical genetics in Africa. Nature, 1–16, doi: papers2://publication/doi/10.1038/nature13997 (2014). 10.1038/nature13997 PMC4297536 25470054

[b2] Barnes K. C., Grant A. V., Hansel N. N., Gao P. & Dunston G. M. African Americans with Asthma: Genetic Insights. Proceedings of the American Thoracic Society 4, 58–68, doi: papers2://publication/doi/10.1513/pats.200607-146JG (2007). 17202293 10.1513/pats.200607-146JG PMC2647616

[b3] Bhangale T. R., Rieder M. J. & Nickerson D. A. Estimating coverage and power for genetic association studies using near-complete variation data. Nat Genet 40, 841–843, doi: papers2://publication/doi/10.1038/ng.180 (2008). 18568023 10.1038/ng.180

[b4] Mathias R. A. . A continuum of admixture in the Western Hemisphere revealed by the African Diaspora genome. Nature Communications 7, 1–10, doi: papers2://publication/doi/10.1038/ncomms12522 (2016). 10.1038/ncomms12522 PMC5062574 27725671

[b5] Ha N.-T., Freytag S. & Bickeboeller H. Coverage and efficiency in current SNP chips. 1–7, doi: papers2://publication/doi/10.1038/ejhg.2013.304 (2014). 10.1038/ejhg.2013.304 PMC4135415 24448550

[b6] Jiang L., Willner D., Danoy P., Xu H. & Brown M. A. Comparison of the performance of two commercial genome-wide association study genotyping platforms in Han Chinese samples. G3 (Bethesda) 3, 23–29, doi: papers2://publication/doi/10.1534/g3.112.004069 (2013). 23316436 10.1534/g3.112.004069 PMC3538340

[b7] Li M., Li C. & Guan W. Evaluation of coverage variation of SNP chips for genome-wide association studies. Eur J Hum Genet 16, 635–643, doi: papers2://publication/doi/10.1038/sj.ejhg.5202007 (2008). 18253166 10.1038/sj.ejhg.5202007

[b8] Nothnagel M., Ellinghaus D., Schreiber S., Krawczak M. & Franke A. A comprehensive evaluation of SNP genotype imputation. Hum. Genet. 125, 163–171, doi: papers2://publication/doi/10.1007/s00439-008-0606-5 (2008). 19089453 10.1007/s00439-008-0606-5

[b9] Johnston H. R., Hu Y. & Cutler D. J. Population Genetics Identifies Challenges in Analyzing Rare Variants. Genet. Epidemiol. 39, 145–148, doi: 10.1002/gepi.21881 (2015). 25640419 PMC4366269

[b10] Li Y., Willer C. J., Ding J., Scheet P. & Abecasis G. R. MaCH: using sequence and genotype data to estimate haplotypes and unobserved genotypes. Genet. Epidemiol. 34, 816–834, doi: papers2://publication/doi/10.1002/gepi.20533 (2010). 21058334 10.1002/gepi.20533 PMC3175618

[b11] Howie B., Fuchsberger C., Stephens M., Marchini J. & Abecasis G. R. Fast and accurate genotype imputation in genome-wide association studies through pre-phasing. Nature Publishing Group 44, 955–959, doi: papers2://publication/doi/10.1038/ng.2354 (2012). 10.1038/ng.2354 PMC3696580 22820512

[b12] Abecasis G. R. & Wigginton J. E. Handling marker-marker linkage disequilibrium: pedigree analysis with clustered markers. Am. J. Hum. Genet. 77, 754–767, doi: papers2://publication/doi/10.1086/497345 (2005). 16252236 10.1086/497345 PMC1271385

[b13] Qin Z. S., Gopalakrishnan S. & Abecasis G. R. An efficient comprehensive search algorithm for tagSNP selection using linkage disequilibrium criteria. Bioinformatics 22, 220–225, doi: papers2://publication/doi/10.1093/bioinformatics/bti762 (2006). 16269414 10.1093/bioinformatics/bti762

[b14] Zheng X. . HIBAG[mdash]HLA genotype imputation with attribute bagging. Pharmacogenomics J 14, 192–200, doi: 10.1038/tpj.2013.18 (2014). 23712092 PMC3772955

[b15] Li H. . The Sequence Alignment/Map format and SAMtools. Bioinformatics 25, 2078–2079, doi: papers2://publication/doi/10.1093/bioinformatics/btp352 (2009). 19505943 10.1093/bioinformatics/btp352 PMC2723002

[b16] Danecek P. . The variant call format and VCFtools. Bioinformatics 27, 2156–2158, doi: papers2://publication/doi/10.1093/bioinformatics/btr330 (2011). 21653522 10.1093/bioinformatics/btr330 PMC3137218

