# Supplementary material for: Identifying tagging SNPs for African specific genetic variation from the African Diaspora Genome
Source: Sci Rep. 2017 Apr 21;7:46398. doi: 10.1038/srep46398 (PMC5399604; doi:10.1038/srep46398)

Identifying tagging SNPs for African specific genetic variation from the African Diaspora Genome

Henry Richard Johnston[1], Yi-Juan Hu[1], Jingjing Gao[2], Timothy D. O'Connor[3,4,5], Goncalo Abecasis[6], Genevieve L Wojcik[7], Christopher R. Gignoux[7], Pierre-Antoine Gourraud[8], Antoine Lizee[8], Mark Hansen[9], Rob Genuario[9], Dave Bullis[9], Cindy Lawley[9], Eimear E. Kenny[7, 10], Carlos Bustamante[7], Terri H. Beaty[11], Rasika A. Mathias[11,12], Kathleen C. Barnes[11,12]* and Zhaohui Steve Qin[1], on behalf of the CAAPA Consortium[12]†

This PDF file contains:

Supplementary Results

Figures S1-S3

Tables S1-S5

The minor allele frequencies (MAF) between sites with African American samples correlate extremely well [Table S1, Figure S1]. As expected, non-African American samples have different MAF spectra. The combined MAF spectrum, however, looks as expected for the ADPC array [Figure S2]. Additionally, we see below-average MAFs for the annotation categories of exonic variants and splice site variants, which is also expected. [Table S2]

The design score spectrum for ADPC variants is strong, suggesting these variants are expected to work well and consistently in terms of making accurate genotype calls, creating a very useful product. [Figure S3] Illumina estimates greater than 85% of variants on the ADPC will consistently generate valid calls. Variants with a design score threshold greater than 0.8 were successful 91.6% of the time on the ADPC.

In examining the classes of genetic variation on the ADPC, the vast majority of variants are either intronic (35%) or intergenic (57%). Among the potentially functional variants, 4410 exonic SNPs are on the array, split nearly evenly between synonymous and non-synonymous variants. [Table S3] Consequently, 70% of variants are classified by ENCODE as heterochromatic. [Table S4] These are chromosomal regions unlikely to be directly involved in transcriptional processes because actively transcribing genes are generally found in euchromatin. The next largest ENCODE category is Weak Transcription, containing 11% of the ADPC variants. Other ENCODE categories include 2% of variants classified as Strong Enhancers, while 3.5% are classified as Weak Enhancers. All of these numbers are consistent with a selection model that focused solely on tagging novel variation, rather than placing functional variants on the array.

Prior to generating whole genome coverage statistics, we examined the statistics for chromosome 22 [Table S5]. The results are similar to, but slightly worse than the whole genome statistics. This is a performance increase of about 10% at each r^2 threshold at the genome-wide level suggests that although the LD block structure in African individuals consists of fairly small blocks, there is some tagging benefit at longer ranges on larger chromosomes, as the ratio of tagSNPs to chromosome length remains fairly constant across the entire genome.

SUPPLEMENTARY TABLES

Supplementary Table S1: Correlations between minor allele frequencies in CAAPA populations in table form

Table S1	Chicago	Detroit	Jackson	HapMap	JHU	UCSF	NIH	W/S
Chicago	NA	0.995	0.995	0.852	0.994	0.944	0.996	0.994
Detroit	0.995	NA	0.997	0.859	0.996	0.95	0.997	0.995
Jackson	0.995	0.997	NA	0.851	0.997	0.944	0.998	0.995
HapMap	0.852	0.859	0.851	NA	0.84	0.943	0.857	0.856
JHU	0.994	0.996	0.997	0.84	NA	0.936	0.997	0.994
UCSF	0.944	0.95	0.944	0.943	0.936	NA	0.949	0.947
NIH	0.996	0.997	0.998	0.857	0.997	0.949	NA	0.996
W/S	0.993	0.995	0.995	0.856	0.994	0.947	0.996	NA

Supplementary Table S2: Minor allele frequency for ADPC variants broken down by annotation category. Splice sites and exonic variants have lower MAFs than other categories

Table S2		
MAF by Annotation Category		
**Category**	**Mean**	**Standard Deviation**
Upstream	0.087	0.115
Downstream	0.098	0.124
Exonic	0.073	0.105
Intronic	0.095	0.122
Intergenic	0.095	0.123
Splicing	0.073	0.079
ncRNA_exonic	0.089	0.117
ncRNA_intronic	0.091	0.122

Supplementary Table S3: Genomic location of ADPC variants

Table S3		
**Sequence Location**	**Count**	**Fraction**
Downstream	5119	0.008
Exonic	4410	0.007
Exonic;splicing	2	3.18E-6
Intergenic	360661	0.575
Intronic	219342	0.35
ncRNA_exonic	2177	0.0035
ncRNA_intronic	22735	0.036
ncRNA_splicing	14	2.23E-5
ncRNA_UTR3	120	0.00019
ncRNA_UTR5	16	2.55E-5
Splicing	24	3.82E-5
Upstream	5464	0.0087
Upstream;downstream	174	0.00028
UTR3	5902	0.0094
UTR5	1376	0.0022

Supplementary Table S4: Predicted variant categories for ADPC variants

Table S4		
**HMM Predicted Category**	**Count**	**Fraction**
Active Promoter	5972	0.0096
Weak Promoter	4957	0.008
Poised Promoter	1989	0.003
Strong Enhancer	12201	0.02
Weak Enhancer	21284	0.034
Insulator	4778	0.008
Txn_Transition	4046	0.007
Txn_Elongation	26169	0.042
Weak_Txn	68178	0.11
Repressed	29272	0.047
Heterochromatin	431507	0.7
Repetitive/CNV	8728	0.014

Supplementary Table S5: Predicted coverage for the ADPC, with and without OmniExpress pairing, on Chromosome 22 alone

Table S5	Coverage of CAAPA variants >= 1% MAF			
	**r^2**	**OmniExpress Alone**	**ADPC Alone**	**Combined**
Chromosome 22	0.9	17%	11%	26%
	0.8	23%	14%	33%
	0.5	34%	28%	51%

SUPPLEMENTARY FIGURES

Supplementary Fig. S1: Scatter plots showing the correlations in minor allele frequency between different populations in CAAPA. African American populations are generally well correlated, while Puerto Rico, Barbados, and Yoruba are all somewhat disparate.

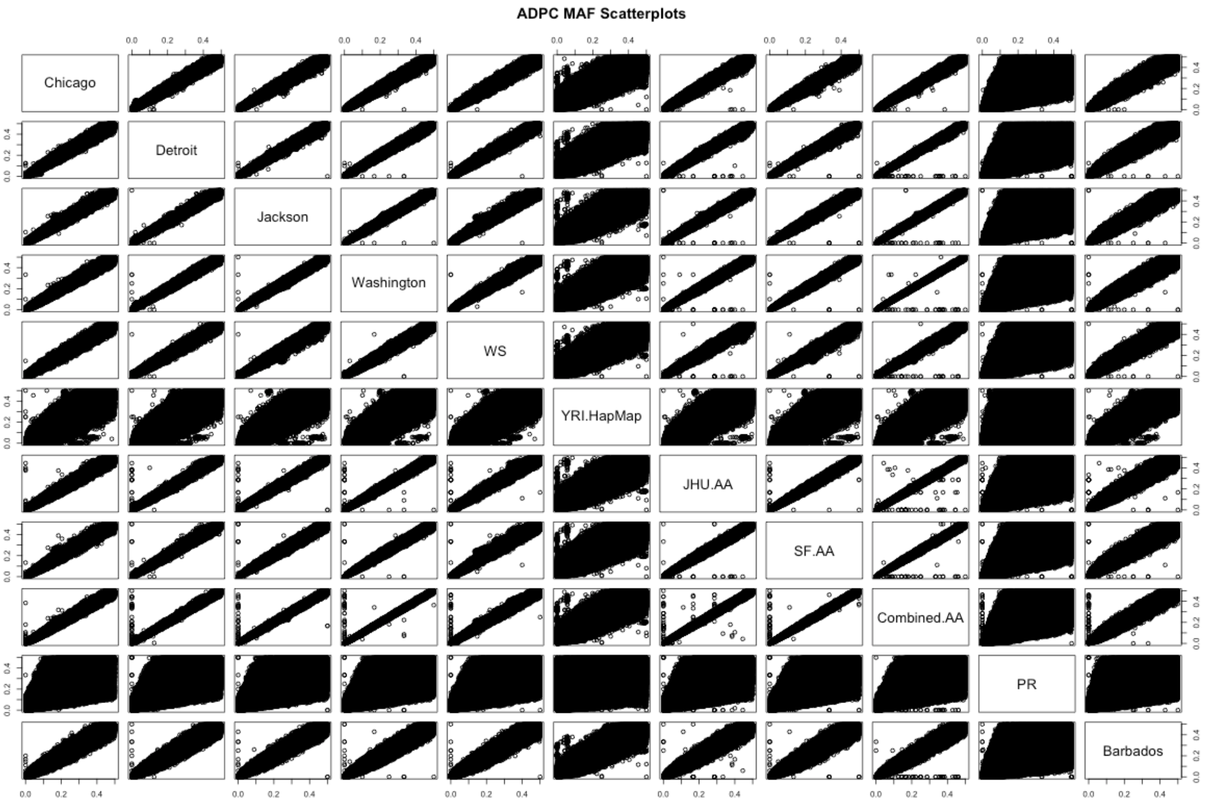

Supplementary Fig. S2: Actual minor allele frequency spectrum of successful markers on the ADPC. It lines up perfectly with the expected frequency spectrum.

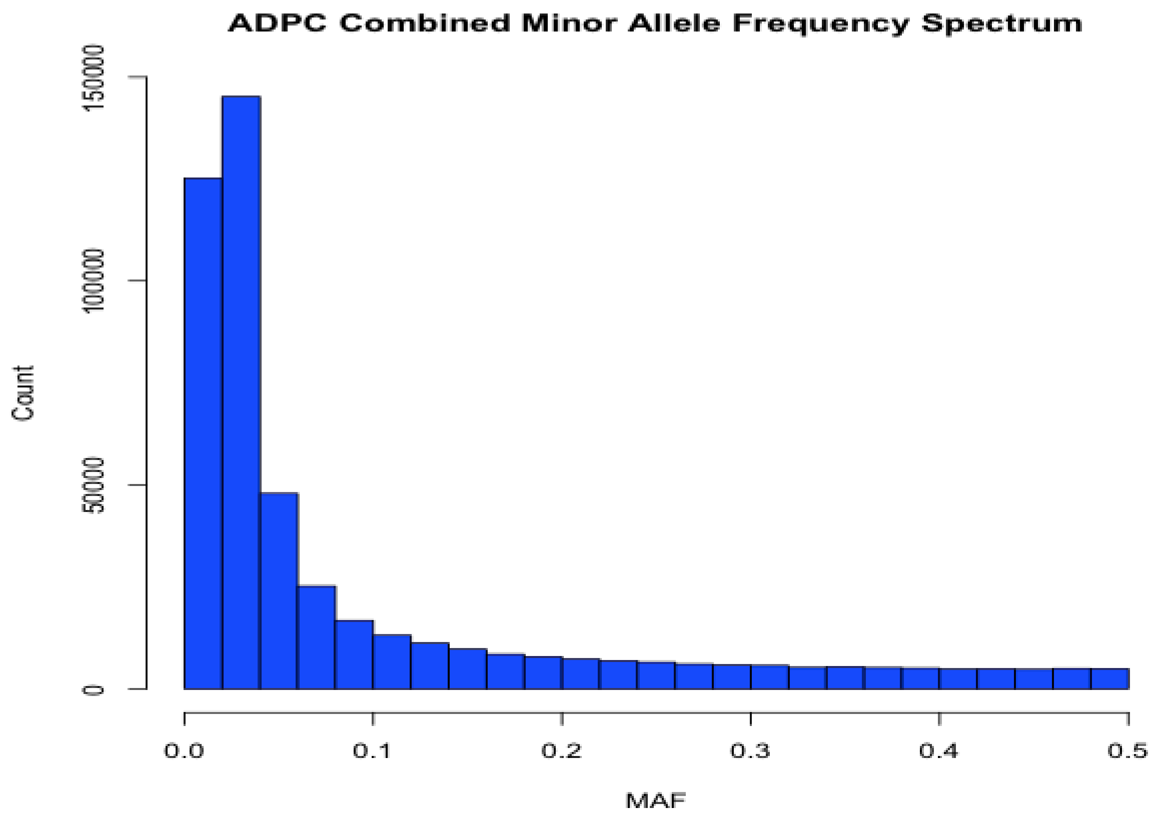

Supplementary Fig. S3: Histogram of Illumina design scores for variants on the ADPC

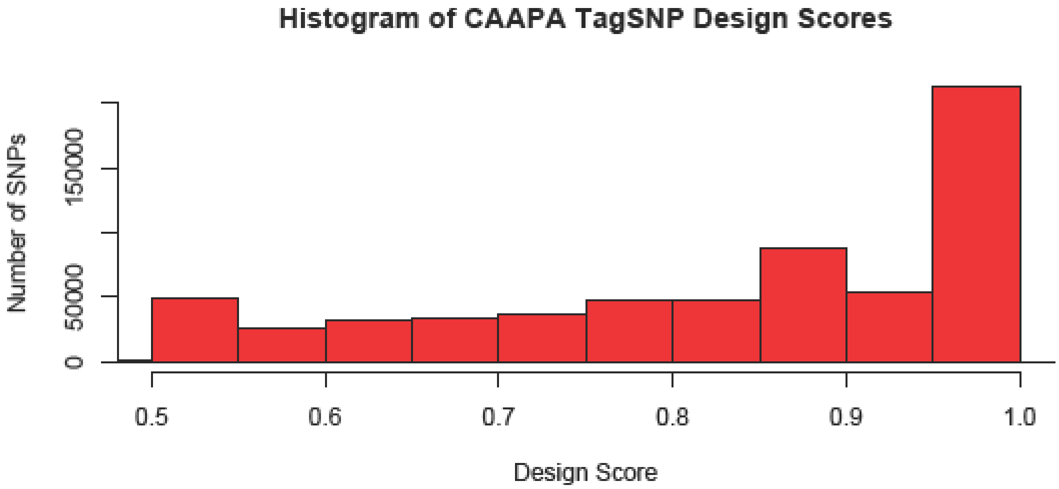

Supplement: Supplementary Information [file srep46398-s1.pdf]
